# Age-Related Changes in Hepatic Activity and Expression of Detoxification Enzymes in Male Rats

**DOI:** 10.1155/2013/408573

**Published:** 2013-07-22

**Authors:** Erika Vyskočilová, Barbora Szotáková, Lenka Skálová, Hana Bártíková, Jitka Hlaváčová, Iva Boušová

**Affiliations:** Department of Biochemical Sciences, Charles University in Prague, Faculty of Pharmacy in Hradec Králové, Heyrovského 1203, CZ-500 05 Hradec Králové, Czech Republic

## Abstract

Process of aging is accompanied by changes in the biotransformation of xenobiotics and impairment of normal cellular functions by free radicals. Therefore, this study was designed to determine age-related differences in the activities and/or expressions of selected drug-metabolizing and antioxidant enzymes in young and old rats. Specific activities of 8 drug-metabolizing enzymes and 4 antioxidant enzymes were assessed in hepatic subcellular fractions of 6-week-old and 21-month-old male Wistar rats. Protein expressions of carbonyl reductase 1 (CBR1) and glutathione *S*-transferase (GST) were determined using immunoblotting. Remarkable age-related decrease in specific activities of CYP2B, CYP3A, and UDP-glucuronosyl transferase was observed, whereas no changes in activities of CYP1A2, flavine monooxygenase, aldo-keto reductase 1C, and antioxidant enzymes with advancing age were found. On the other hand, specific activity of CBR1 and GST was 2.4 folds and 5.6 folds higher in the senescent rats compared with the young ones, respectively. Interindividual variability in CBR1 activity increased significantly with rising age. We suppose that elevated activities of GST and CBR1 may protect senescent rats against xenobiotic as well as eobiotic electrophiles and reactive carbonyls, but they may alter metabolism of drugs, which are CBR1 and especially GSTs substrates.

## 1. Introduction

Aging is an inevitable biological process characterized by progressive functional decline of all organ systems with simultaneous increase in oxidative damage as a result of reactive oxygen species (ROS) accumulation. In general, activity of various enzymes is lower in very old individuals than in the period of adulthood [1]. Important changes with age, which can affect the pharmacokinetics of a drug, are changes in body composition with an increase in body fat and a decrease in body water, decrease in kidney and liver (20%–50%) blood flow reducing renal excretion and possibly elimination of high-extraction drugs, and a reduction in liver mass by 20%–30%, which can result in reduced metabolism rates [2, 3].

With increasing age, a decline of biotransformation capacity was observed in laboratory animals and in man [4]. In old animals and humans, a marked fall in cytochromes P450 (CYP) activities and drugs oxidative biotransformation has been repeatedly described (e.g., [5, 6]). However, information about age-related differences in activities of other biotransformation enzymes has been limited. These enzymes, such as flavine monooxygenases (FMOs), carbonyl reductases (CBRs), aldo-keto reductases (AKRs), glutathione S-transferases (GSTs), and UDP-glucuronosyl transferases (UGTs), play also an important role in drug metabolism, and age-related alterations in their activity in humans may have important clinical consequences by affecting both drug efficacy and toxicity [7]. 

In detoxification of organism, not only biotransformation enzymes but also antioxidant enzymes participate. While biotransformation enzymes generally protect organisms against potentially toxic xenobiotics, antioxidant enzymes defend an organism against the reactive oxygen species (ROS). Aerobic cells produce ROS, for example, superoxide anion radical (O_2_^•−^), hydrogen peroxide (H_2_O_2_), and hydroxyl radical (HO^•^), as the by-products of their metabolic processes. The ROS cause oxidative damage to macromolecules under conditions in which the antioxidant defense of the body is overwhelmed [8–10]. All aerobic organisms require mechanisms that limit molecular damage caused by eliminating ROS. The antioxidant enzymes (e.g., superoxide dismutase, catalase, and glutathione peroxidase) are essential parts of this antioxidant defense system. Catalase (CAT) decomposes H_2_O_2_ to H_2_O and O_2_. Superoxide dismutase (SOD) catalyzes the dismutation of superoxide anions to hydrogen peroxide [11]. Nonspecific peroxidases (Px) are responsible for the reduction of hydrogen peroxide or organic hydroperoxides, such as lipid peroxides. Selenium-dependent glutathione peroxidase (GPx) catalyzes the reduction of H_2_O_2_ to H_2_O at the expense of reduced glutathione (GSH) [12]. GPx can also remove organic hydroperoxides. During aging, the accumulation of deleterious effects of ROS was observed. A certain amount of oxidative damage takes place even under normal conditions; however, the rate of this damage increases during the aging process as the efficiency of antioxidant and repair mechanisms decreases [13–15].

The aim of the present work was to study age-related changes in the activities and expression of selected xenobiotic-metabolizing enzymes (CYP1A2, CYP2B, CYP3A, FMO, CBR1, AKR1C, GST, and UGT) and some antioxidant enzymes (CAT, SOD, GPx, and Px) in rat liver.

## 2. Materials and Methods

### 2.1. Chemicals and Reagents

Menadione, 7-methoxyresorufin, 7-pentoxyresorufin, benzyloxyresorufin, 1-chloro-2,4-dinitrobenzene (CDNB), reduced GSH, rabbit polyclonal anti-CBR1 antibody, Triton-X, ethylenediaminetetraacetic acid, thiobenzamide, glutathione reductase, UDP-glucuronic acid, glucose-6-phosphate dehydrogenase, glucose-6-phosphate, o-phenylenediamine, acenaphthenol, trichloroacetic acid, and chemicals used for realization of electrophoresis were products of Sigma-Aldrich (Prague, Czech Republic). Coenzymes NADPH and NADP^+^ were supplied by Merck (Prague, Czech Republic). Superoxide dismutase (SOD) Assay Kit-WST (Dojindo, Tabaru, Japan) was purchased from Probior (Munich, Germany). Bovine serum albumin (BSA), 4-nitrophenol, and *tert*-butyl hydroperoxide were obtained from Fluka (Prague, Czech Republic). Precision plus molecular weight standard and nonfat dry milk were products of Bio-Rad (Hercules, USA). Goat polyclonal anti-GSTA antibody, mouse monoclonal anti-beta actin antibody, rabbit polyclonal anti-goat IgG antibody conjugated with alkaline phosphatase, rabbit polyclonal anti-mouse IgG antibody conjugated with alkaline phosphatase, and goat polyclonal anti-rabbit IgG antibody conjugated with alkaline phosphatase were purchased from Abcam (Cambridge, UK). All other chemicals used were of HPLC or analytical grade.

### 2.2. Animals

Male Wistar rats were obtained from BioTest (Konarovice, Czech Republic). They were housed in air-conditioned animal quarters with a 12 h light/dark cycle. Food (a standard rat chow diet) and water were provided *ad libitum*. The rats were cared for and used in accordance with the Guide for the Care and Use of Laboratory Animals (Protection of Animals from Cruelty Act No. 246/92, Czech Republic). Groups of 6-week-old and 21-month-old rats, each consisting of six animals, were used. At the end of experiment, animals were sacrificed by decapitation; liver tissues were removed immediately and stored at −80°C until preparation of subcellular fractions.

### 2.3. Preparation of Subcellular Fractions and Protein Content Determination

Liver tissues of about 5 g were individually homogenized in 0.1 M sodium phosphate buffer (pH 7.4) using a Potter-Elvehjem homogenizer. The subcellular fractions were isolated by differential centrifugation of the tissue homogenate [16] and stored at −80°C.

The cytosolic fractions (the supernatant after ultracentrifugation at 105 000 ×g for 1 h) were preserved in aliquots at −80°C until further analyses. The microsomal fractions (protein pellets after additional ultracentrifugation at 105 000 ×g for 1 h) were resuspended in a buffer containing 20% glycerol (v/v), and aliquots were stored at −80°C until further analyses. Protein concentration was determined by the bicinchoninic acid assay [17] using bovine serum albumin as a standard.

### 2.4. Enzyme Assay

Each enzyme assay was performed at least in triplicate for each animal. 

The cytochrome P450 (CYP1A2, 2B, and 3A), 7-methoxyresorufin (MROD), 7-penthoxyresorufin (PROD), and benzyloxyresorufin O-dealkylase (BROD) activities were determined using fluorimetric determination of resorufin at 37°C [18]. Each substrate (dissolved in dimethylsulfoxide, DMSO) was added at a final concentration of 2.5 *μ*M. The amount of microsomal protein in the reaction mixture ranged between 0.30 and 0.35 mg. Assays were conducted using a Perkin-Elmer luminescence spectrophotometer LS 50B, *λ*_EX_/*λ*_EM_ = 530 nm/585 nm. The product formation was monitored continuously for 3 minutes. The MROD, PROD, and BROD activities were calculated using the standard amount-addition technique. The total CYP content was determined according to the method of Omura and Sato [19]. Sodium dithionate was added to the liver microsomes, and the reduced hemoprotein was combined with carbon monoxide by bubbling CO through the solution. The characteristic absorbance at 450 nm was determined spectrophotometrically.

The activity of flavine monooxygenases was determined using thiobenzamide S-oxidation at 35°C [20]. The reaction mixture contained 1 mM thiobenzamide dissolved in acetonitrile, 20 *μ*L of microsomes, and 180 *μ*L of NADPH-regenerating system. Formation of thiobenzamide S-oxide was assessed spectrophotometrically at 370 nm by Tecan Infinite M200.

The activity of carbonyl reductase 1 was assayed using 50 *μ*M menadione (dissolved in ethanol) [21]. The amount of organic solvent in the final reaction mixtures did not exceed 0.5% (v/v). Spectrophotometric determination (340 nm, 37°C) of NADPH (100 *μ*M) consumption served as the assessment of CBR1 activities. Assays were performed using the spectrophotometer Helios ß (Spectronic Unicam). 

The enzyme activity for a model substrate 1-acenaphthenol (substrate of AKR1C) was determined using the method described by Palackal et al. [22] with some modifications. The velocity of substrate dehydrogenation was determined spectrophotometrically by measuring the change in absorbance caused by reduction of the oxidized cofactor NADP^+^ at 340 nm. The final 1.0 mL system contained 1 mM acenaphthenol dissolved in DMSO (1% of organic solvent in final mixture), 1.0 mM NADP^+^, 50 *μ*L of cytosol, and 0.1 M Tris-HCl buffer (pH 8.9).

The cytosolic glutathione S-transferase activity was assessed by standard colorimetric assay using 1-chloro-2,4-dinitrobenzene as an electrophilic substrate (dissolved in ethanol) [23]. The reaction mixture (total volume of 1.0 mL) contained 10 *μ*L of cytosol (0.13–0.18 mg of protein), 1 mM reduced glutathione, and 1 mM CDNB in 0.1 M Na-phosphate buffer (pH 6.5). The absorbance of the rising product S-(2,4-dinitrophenyl) glutathione was detected at 340 nm using the spectrophotometer Helios ß (Spectronic Unicam).

The microsomal UDP-glucuronosyl transferase activity was assayed by the method of  Mizuma et al. [24]. Microsomes were preincubated with Slovasol detergent at 4°C for 20 min. The reaction mixture (total volume of 0.1 mL) contained 10 *μ*L of microsomes (0.12–0.14 mg of protein), 0.33 mM UDP-glucuronic acid, and 166.8 *μ*M *p*-nitrophenol (dissolved in redistilled water) in 0.1 M Tris/HCl buffer (pH 7.4). After 20 min of incubation at 37°C, the absorbance was measured at 415 nm using Tecan Infinite M200.

Catalytic activity of catalase was assessed by the spectrophotometric method according to Goth [25]. Twenty *μ*L of cytosol was incubated with 100 *μ*L of hydrogen peroxide (65 mM hydrogen peroxide in 60 mM Na/K-phosphate buffer, pH 7.4) at 37°C for 60 s. The enzymatic reaction was stopped with 100 *μ*L of 32.4 mM ammonium molybdate, and the yellow complex of molybdate and hydrogen peroxide was measured at 405 nm using Tecan Infinite M200. 

The assay of superoxide dismutase was performed using SOD Assay Kit-WST according to the general protocol. This enables measurement by utilizing the highly water-soluble tetrazolium salt WST-1 [2-(4-iodophenyl)-3-(4-nitrophenyl)-5-(2,4-disulfophenyl)-2H-tetrazolium, monosodium salt], which produces water-soluble formazan upon reduction with the superoxide anion radical (O_2_^•−^). Production of O_2_^•−^ is generated by the xanthine oxidase and is inhibited by SOD. Therefore, the inhibition activity of SOD on O_2_^•−^ formation leads to lower formazan production, which can be determined by a colorimetric method. 

Enzyme activity of glutathione peroxidase was measured by a coupled reaction with glutathione reductase (GR). In the assay, glutathione is oxidized to glutathione disulfide (GSSG) by the GPx-mediated reduction of *tert*-butyl hydroperoxide. Subsequent GSSG reduction caused by GR is accompanied by the concomitant oxidation of NADPH, resulting in measurable rates of decrease in absorbance at 340 nm (Tecan Infinite M200) [26, 27].

Nonspecific peroxidase activity was determined by the method based on the oxidation of o-phenylenediamine dihydrochloride in the presence of H_2_O_2_ [28]. The output 2,2′-diaminoazobenzene was detected spectrophotometrically with a microplate reader (Tecan Infinite M200) at 490 nm.

### 2.5. Western Blotting

Prepared cytosolic fractions from liver samples were used to perform western blotting. Analyses of individual samples (obtained from 6 young and 6 old rats) and two pooled samples (each containing a mixture of 6 cytosolic fractions *ana partes aequales*) were performed. Separation of proteins was carried out on Mini-Protean III system (Bio-Rad, Hercules, CA, USA) by sodium dodecyl sulfate-polyacrylamide gel electrophoresis (SDS-PAGE) using discontinuous system with 4% stacking gel and 12.5% separating gel according to the method of Laemmli [29]. Lanes were loaded with 10 *μ*g of proteins. The proteins were then transferred onto the nitrocellulose membrane (0.45 *μ*m, Bio-Rad) in transfer buffer (25 mM Tris, 190 mM glycine, and 20% methanol) using a wet blotting procedure [30]. Blotting was performed at a constant voltage 100 V for 90 min (Mini Trans-Blot Electrophoretic Transfer Cell, Bio-Rad). After blotting, membranes were blocked in 8% nonfat dry milk in Tris/HCl-buffered saline and Tween-20 (TBST) buffer overnight at 4°C. All further incubations were carried out at room temperature on a platform shaker. After washing in TBST buffer, the membranes were treated with primary antibodies (goat polyclonal anti-GSTA antibody; rabbit polyclonal anti-CBR1 antibody) for 60 min at room temperature. Subsequently, membranes were washed six times with TBST buffer and incubated with alkaline phosphatase-conjugated corresponding secondary antibodies (Abcam, Cambridge, UK) for 60 min. The blots were extensively washed in 0.1 M Tris/HCl buffer containing 5 mM MgCl_2_·6H_2_O (pH 9.5), covered with chemiluminescent substrate DuoLux for 5 min, and then exposed to X-ray film (CL-XPosure Film, Thermo Fisher Scientific). Specific protein bands on the nitrocellulose membranes were quantified densitometrically (Quantity One software, Bio-Rad, Hercules, CA, USA). Beta-actin (mouse monoclonal anti-beta-actin antibody, Abcam, Cambridge, UK) served as a loading control.

### 2.6. Statistical Analysis

All calculations were done using Microsoft Excel and GraphPad Prism 5.04. All values were expressed as mean ± SD. Student's *t*-test was used for the statistical evaluation of differences between young and old rats, and differences were regarded as significant when *P* < 0.01.

## 3. Results

 In the present study, catalytic activities and protein expression of selected biotransformation and antioxidant enzymes were assessed in subcellular fractions (cytosol and microsomes) isolated from livers of 6-week-old (juniors) and 21-month-old (seniors) male rats. The average of life span of male Wistar rats is almost 25 months [31]. At the time rats were sacrificed, the average body weight of the juniors and seniors was 233  ±  20.7 and 700  ±  79.3 g, respectively. Also the average liver weight remarkably increased with age (10.9  ±  0.7 versus 20.5  ±  4.0 g).

**Figure 1:**
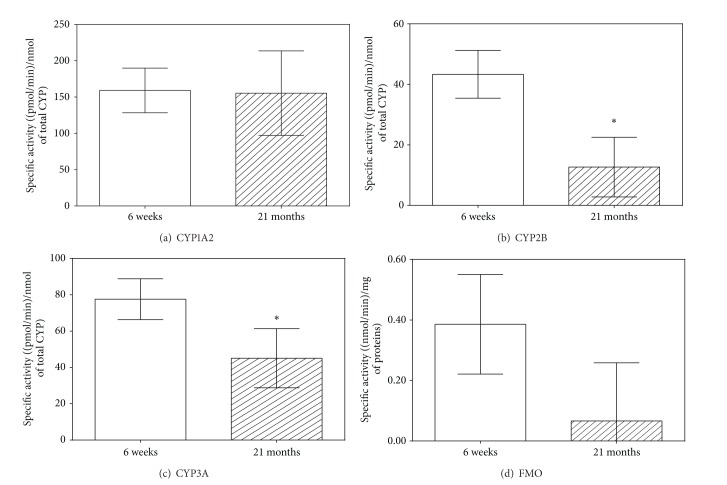
Age-related differences in specific activities of biotransformation enzymes with oxidizing activity in rat liver. The white column represents young (6-week-old) rats, and the hatched column indicates old (21-month-old) rats. The activities of individual CYP isoforms were normalized to the total CYP content. Data of each age group represent the mean ± SD (six animals per age group) (*data with *P* < 0.01, Student's *t*-test). CYP = cytochrome P450; FMO = flavine monooxygenase.

*The Phase I Oxidative Enzymes*. The specific activities of CYP (isoforms CYP1A2, CYP2B, and CYP3A) normalized to the total CYP content and specific activity of FMO were assessed in microsomal fraction of rat liver (Figure 1). Total CYP content decreased significantly with advancing age (1.03  ±  0.03 *μ*M versus 0.83  ±  0.03 *μ*M). Catalytic activities of CYP2B and CYP3A significantly decreased with increasing age of experimental animals. While specific activity of CYP2B was 3.4 times higher in the rat juniors compared with the seniors (Figure 1(b)), CYP3A activity in the 6-week-old rats was only 1.7 times higher than in the 21-month-old rats (Figure 1(c)). FMO activity also decreased in the old rats, but this decline was not statistically significant (Figure 1(d)). Nonsignificant decline in CYP1A2 activity in the old rats with respect to the young rats was observed too (Figure 1(a)).

**Figure 2:**
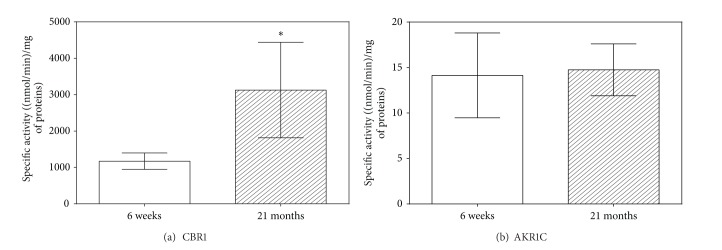
Specific activities of biotransformation enzymes with reducing activity in rat liver. The white column represents young (6-week-old) rats, and the hatched column indicates old (21-month-old) rats. Values are expressed as mean ± SD (six animals per age group) (*data with *P* < 0.01, Student's *t*-test). CBR1 = carbonyl reductase 1; AKR1C = aldo-keto reductase 1C.

*The Phase I Reducing Enzymes*. Activities of CBR1 and AKR1C were tested in cytosolic fractions of rat liver homogenates. Activity of CBR1, assayed using menadione as a specific substrate, was elevated in the old rats by 166.7% compared with the young rats (Figure 2(a)). No age-related changes in the activity of AKR1C, assessed using a model substrate 1-acenaphthenol, were observed (Figure 2(b)).

**Figure 3:**
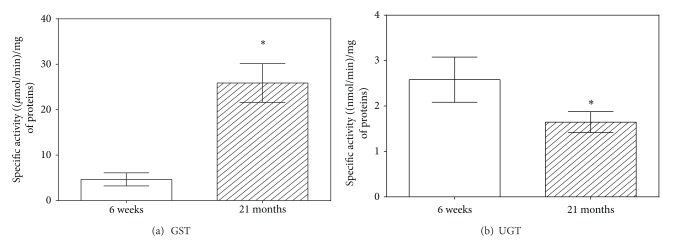
Specific activities of biotransformation enzymes with conjugative activity in rat liver. The white column represents young (6-week-old) rats, and the hatched column indicates old (21-month-old) rats. Values are expressed as mean ± SD (six animals per age group) (*data with *P* < 0.01, Student's *t*-test). GST = glutathione *S*-transferase; UGT = UDP-glucuronosyl transferase.

*The Phase II Conjugating Enzymes*. Specific activities of GST and UGT were tested in cytosolic and microsomal fractions of rat liver homogenates, respectively. Catalytic activity of GST was assessed by measuring its conjugation activity with the universal substrate CDNB. GST activity was increased 5.6 times in the group of old rats compared with the group of young rats (Figure 3(a)). Activity of UGT, assayed using *p*-nitrophenol as a substrate, was significantly decreased in the 21-month-old rats than in the 6-week-old rats (Figure 3(b)).

**Figure 4:**
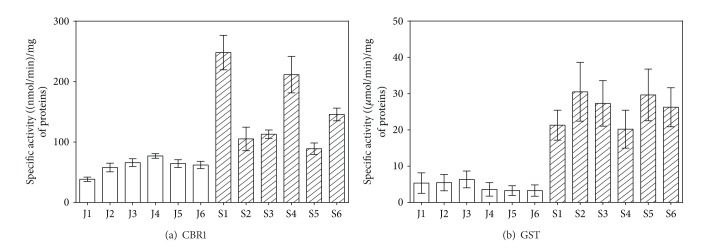
Interindividual differences in specific activities of GST and CBR1 in rat liver. The white columns represent 6-week-old rats (J1–J6), and hatched columns indicate 21-month-old rats (S1–S6). Values are expressed as mean ± SD (six animals per age group). GST = glutathione *S*-transferase; CBR1 = carbonyl reductase 1.

*The Interindividual Differences in CBR1 and GST Activities*. Differences in the level of specific activity of CBR1 and GST, whose activities rose with age, between individuals of both age groups were observed. In both enzymes, differences in the absolute values of catalytic activity between individuals increased with age. In CBR1, the activity was in the range of 38.5  ±  3.7 nmol/min/mg and 77.0  ±  3.8 nmol/min/mg in the 6-week-old rats and in the range of 89.0  ±  9.5 nmol/min/mg and 248.1  ±  28.5 nmol/min/mg in the 21-month-old rats (Figure 4(a)). Level of GST activity in the 6-week-old rats ranged between 3.3  ±  1.3 *μ*mol/min/mg and 6.3  ±  2.3 *μ*mol/min/mg, while in the 21-month-old rats it varied from 20.2  ±  5.2 *μ*mol/min/mg to 30.5  ±  8.1 *μ*mol/min/mg (Figure 4(b)).

**Figure 5:**
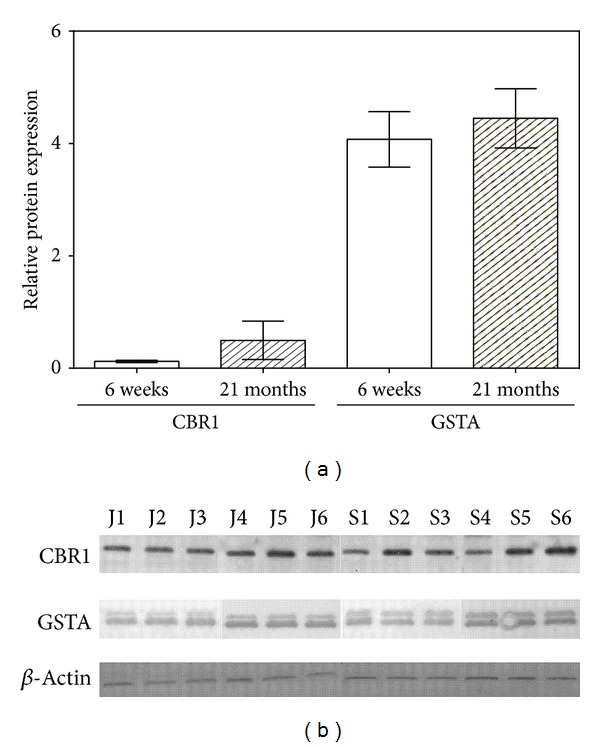
Age-related changes in the hepatic CBR1 and GSTA protein expression normalized to the amount of *β*-actin (a). Interindividual differences in protein expression of GST and CBR1 in rat liver (b). Cytosolic fractions (4 *μ*g of protein/lane) of 6-week-old (J1–J6) and 21-month-old (S1–S6) rats were subjected to SDS-PAGE with subsequent immunoblotting. Blots were then reacted with rabbit polyclonal anti-CBR1 antibody or goat polyclonal anti-GSTA antibody. Protein bands were marked by corresponding secondary antibodies using a chemiluminescent detection with an alkaline phosphatase substrate. Protein expression was normalized to the amount of the loading control *β*-actin using mouse monoclonal anti-beta-actin antibody. GSTA = alpha-glutathione *S*-transferase; CBR1 = carbonyl reductase 1.

*Expression of CBR1 and GST*. The amounts of CBR1 protein and GSTA protein were detected by immunoblotting both in the individual samples and in the pooled samples. Insignificant increase in the levels of CBR1 and GSTA protein normalized to the amount of constitutive protein (*β*-actin) was found in the group of old rats compared with the group of young rats (Figure 5(a)). However, interindividual differences in protein expression of CBR1 as well as GSTA between individuals of both age groups were observed (Figure 5(b)).

**Figure 6:**
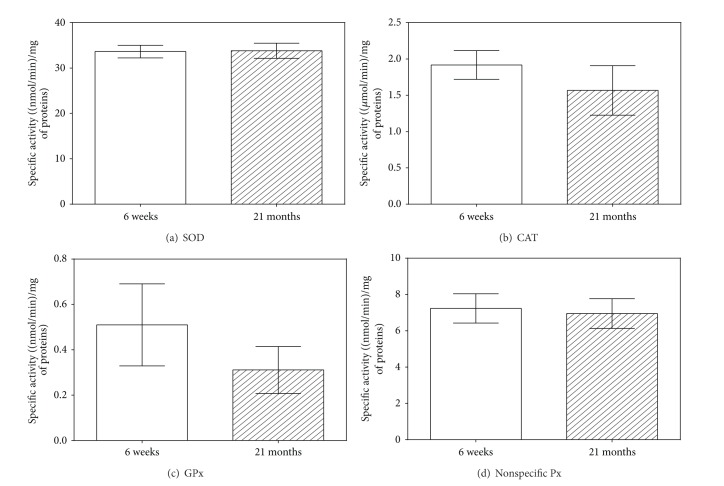
Age-related differences in specific activities of antioxidant enzymes in rat liver. The white column represents young (6-week-old) rats, and the hatched column indicates old (21-month-old) rats. Values are expressed as mean ± SD (six animals per age group) (*data with *P* < 0.01, Student's *t*-test). SOD = superoxide dismutase; CAT = catalase; GPx = glutathione peroxidase; nonspecific Px = nonspecific peroxidase.

*The Antioxidant Enzymes*. Specific activities of SOD, CAT, GPx, and nonspecific Px were tested in cytosolic fractions of rat liver homogenates. No age-related changes in the activities of SOD, CAT, and nonspecific Px were observed (Figures 6(a), 6(b), and 6(d)). GPx activity was reduced by 39.0% in 21-month-old rats compared with the 6-week-old rats, although this decrease was not statistically significant (Figure 6(c)).

## 4. Discussion

In vertebrates, the liver represents the major site for the metabolic clearance of foreign compounds and is also the richest source of enzymes catalyzing biotransformation of xenobiotics [32]. Expression and activity of hepatic biotransformation enzymes may be significantly changed during development and with age, gender, genetic factors, nutrition, and pathophysiological conditions [6]. Changes in expression of drug-metabolizing enzymes may significantly affect metabolism and biological effects of many drugs, industrials, and environmental contaminants [1]. Nonetheless, the influence of aging on xenobiotic metabolism is still a matter of controversy for humans as well as for rats. To our knowledge, there is no report in the literature which would describe changes in the drug-metabolizing as well as antioxidant enzymes in the tissue samples obtained from the same group of animals. Therefore, this study was designed to compare the activity/expression of selected phase I oxidative (CYP and FMO), phase I reducing (CBR1 and AKR1C), and phase II conjugating enzymes (UGT and GST) in liver of the 6-week-old and the 21-month-old male rats.

In this study, age-related changes in the specific activities of CYP isoforms 1A2, 2B, and 3A assessed using isoform-specific substrates 7-methoxyresorufin, pentoxyresorufin, and benzyloxyresorufin were observed, respectively. Specific activities of CYP2B and CYP3A decreased remarkably in 21-month-old rats, while age-associated decline in CYP1A2-specific activity was not statistically significant. The obtained results are consistent with the previously published results (e.g., [5, 6]). In rats, increasing age caused significant alterations in the expression and activity of CYP isoforms in an isoform-specific manner, suggesting that the CYP-dependent drug-metabolizing system may be fully activated after puberty and decreased with aging (e.g., [6, 33]). While CYP1A2, CYP2B1, and CYP2E1 were maximally expressed at 3-week-old male Sprague-Dawley rats and their expression decreased after puberty (12 weeks), CYP2C11 and CYP3A2 increased considerably after puberty and decreased with aging [6]. Similarly, decline of the CYP3A activity and expression by 50%–70% in the 25-26-month-old male Fisher-344 rats compared with the 3-4-month-old animals was observed [5]. In contrast, no changes in hepatic CYP3A protein as well as CYP3A activity, assessed as midazolam oxidation, were observed in male Wistar rats over the entire age range studied (3–18 months) [34]. Decrease in CYP activity observed in this study may be caused by the age-related loss of smooth endoplasmic reticulum in hepatocytes or changes in cofactor supplies [35, 36].

However, activities of known catalytic markers of human CYPs (e.g., MROD, BROD, and PROD) need not be specific also for rat orthologous forms. BROD activity was also demonstrated to be a specific marker of mouse CYP3A, while it may be catalyzed by CYP3A, CYP1A2, and CYP2B6 in humans [37]. Kobayashi et al. [38] reported that BROD and PROD activities were catalyzed by CYP1A2 and CYP2B1 in rats. The 7-methoxyresorufin appeared not to effectively discriminate CYP1A2 from CYP1A1 or CYP2C6 [37].

The developmental and tissue-specific expression of FMOs has been characterized in a number of animal species, including humans, mice, rats, and rabbits [39]. FMO isoforms exhibit distinct cell-, tissue-, sex-, and developmental stage-specific patterns of expression; the most abundant isoforms (3 and 5) increase during puberty and decline with age, suggesting a physiological role during development [40, 41]. Consistently with these findings, the decrease in FMO-specific activity in 21-month-old rats compared with the 6-week-old individuals was observed in our experiments, although this decrease was not significant due to large interindividual variability. 

Decline in CYP as well as FMO activity/expression with aging may significantly affect biological effects as well as toxicity of drugs and other xenobiotics. The decrease in hepatic CYP content, nicotine oxidase activity, and FMO activity was accompanied by alterations in nicotine acute toxicity in 24-month-old Wistar rats compared with the 6-week-old individuals [42]. 

Results of our experiments revealed for the first time the marked age-related increase in the activity of CBR1, which is involved in the biotransformation of a wide range of xenobiotics containing carbonyl group. In contrast, no changes in the AKR1C activity, assessed using a model substrate 1-acenaphthenol, were found in rat liver during aging. To our knowledge, there are no reports concerning age-related changes of  hepatic CBR1 and AKR1C activities and/or expressions. Only Inazu and Fujii [43] reported that activity of rat testicular carbonyl reductase reached a peak value at 3 weeks, then it steeply declined at 4 weeks, and did not further change between 4 and 20 weeks of age. 

In this presented work, remarkable decline in the activity of UGT in 21-month-old rats compared with the 6-week-old ones was observed. Chengelis [33] studied age- (4 to 103 weeks) and sex-related changes in the UGT activity in the Sprague-Dawley rats. Female rats exerted maximal conjugative activity towards *p*-nitrophenol at week 39, while male rats had greater UGT activity at weeks 12 to 39. These data are in a good agreement with the presented results. On the other hand, glucuronidation of xenobiotics has been reported to be unaffected by aging both in microsomes (using acetaminophen) and in isolated perfused livers (for *p*-nitrophenol) [36, 44]. We hypothesize that the decrease in UGT activity observed in this study may be caused by the loss of smooth endoplasmic reticulum in hepatocytes, reduced hepatic content of substrates for glucuronidation (i.e., glycogen, UDP-glucose, and UDP-glucuronic acid), or changes in glucuronosyl transferase kinetic parameters in senescent rats [35, 36]. 

The key reaction catalyzed by GSTs is the conjugation of electrophilic xenobiotics (e.g., drugs, toxins, environmental pollutants, products of oxidative stress, and carcinogens) with endogenous tripeptide GSH. This conjugation is usually considered as a detoxification procedure. Moreover, some GSTs participate also in the synthesis of prostaglandins and leukotrienes [45]. In this study, almost 6-fold increase in GST activity with age, assessed using universal substrate CDNB, was observed. This tendency was not confirmed by immunoblotting with primary antibody specific to rat alpha-class GST. It is possible that increase in total GST activity may be caused by elevated expression of the GSTs from (an)other class(es). Age-related changes in GST activities in rat livers have been studied in numerous reports, but contradictory results were reported. Age-related decrease of hepatic GST was described by Farahmand et al. [31]; Helmy [46]; and Chengelis [47], whereas no changes of GST were observed by Aydin et al. [48] and Carrillo et al. [49]. On the other hand, elevation in liver and brain GST activities in male Wistar rats during aging have been reported by Kim et al. [50] and Oztürk and Gümüşlü [51]. Also increase in plasma GST activity as a function of human age was described [52]. Elevation in GST activity/expression observed in this study could be explained as a compensatory mechanism against increased formation of oxidative stress products (e.g., organic hydroperoxides) accompanying process of aging. 

One of the factors that may affect outcomes of therapies and detoxification of xenobiotics is the interindividual variability in the expression of xenobiotic enzymes. For example, expression level of a predominant hepatic doxorubicin (DOX) reductase CBR1 and the activity of DOX reduction varied >70- and 22-times in human livers, respectively, but showing no association with CBR1 gene variants found in those samples. Interindividual variability in CBR1 expression may thus affect the antitumor effect and the risk of DOX-induced heart failure [53]. In addition, the most remarkable characteristic of liver function in the elderly is the increase in interindividual variability, a feature that may obscure true age-related differences [35].

Cellular redox state is a consequence of the precise balance between the levels of oxidizing and reducing equivalents, such as ROS and endogenous antioxidants. However, it appears that during the aging process an imbalance between oxidants and antioxidants may occur, referred to as oxidative stress [46]. Antioxidant enzymes constitute one of the major cellular defense mechanisms against various oxidative insults [54]. 

In this study, no changes in hepatic activities of SOD, CAT, GPx as well as nonspecific Px with advancing age were found, which is in a good agreement with the data found in the literature. Several reports describing age-related changes in the activities of antioxidant enzymes have been published. In the case of SOD, conflicting results can be found in the literature because decrease [31, 55], no change [46], as well as increase [50, 56] in hepatic SOD activity have been observed in aging male Wistar rats. Activity of CAT either declined [31, 46] or did not change [50, 55, 56] during aging in livers of male rats. GPx hepatic activity was reported to be elevated in 9-month-old as well as 31-month-old male rats compared with the young animals [50, 55]. On the other hand, Helmy [46] described remarkable decrease in GPx activity in 22-month-old rats compared with the 3-month-old male rats. Oztürk and Gümüşlü [51] described significant rise in SOD and CAT activities, while GPx activity was reduced in erythrocytes of 12-month-old rats compared with the 1-month-old rats. Significant positive correlations between plasma SOD and CAT activities and rising human age were found by Rizvi and Maurya [9].

## 5. Conclusions

In this study, remarkable age-related decrease in specific activities of CYP2B, CYP3A, and UGT was found in male rats. On the other hand, significant elevations in CBR1 as well as GSTs activities were observed in the 21-month-old rats compared with the 6-week-old ones. The more pronounced increase was observed in GST of which specific activity was about 6 folds higher and corresponding protein was insignificantly augmented in 21-month-old rats than in young animals. Interindividual variability in the activity of CBR1, which was observed in both groups, increased with rising age. We suppose that elevated activities of GST and CBR1 may protect senescent rats against xenobiotic as well as eobiotic electrophiles and reactive carbonyls, but they may alter metabolism of drugs, which are CBR1 and especially GSTs substrates. 

## Abbreviations

AKR: Aldo-keto reductase
BROD: Benzyloxyresorufin
CAT: Catalase
CBR: Carbonyl reductase
CDNB: 1-Chloro-2,4-dinitrobenzene
CYP: Cytochrome P450
DMSO: Dimethylsulfoxide
FMO: Flavine monooxygenase
GPx: Glutathione peroxidase
GR: Glutathione reductase
GSH: Glutathione
GSSG: Glutathione disulfide
GST: Glutathione S-transferase
MROD: 7-Methoxyresorufin
PAGE: Polyacrylamide gel electrophoresis
PROD: 7-Penthoxyresorufin
Px: Nonspecific peroxidases
ROS: Reactive oxygen species
SOD: Superoxide dismutase
TBST: Tris/HCl-buffered saline-Tween-20
UGT: UDP-glucuronosyl transferase.

## References

[1] Testa B, Krämer SD (2010). *The Biochemistry of Drug Metabolism: Conjugations, Consequences of Metabolism, Influencing Factors*.

[2] Benedetti MS, Whomsley R, Canning M (2007). Drug metabolism in the paediatric population and in the elderly. *Drug Discovery Today*.

[3] Klotz U (2009). Pharmacokinetics and drug metabolism in the elderly. *Drug Metabolism Reviews*.

[4] Klinger W (2005). Developmental pharmacology and toxicology: biotransformation of drugs and other xenobiotics during postnatal development. *European Journal of Drug Metabolism and Pharmacokinetics*.

[5] Warrington JS, Greenblatt DJ, von Moltke LL (2004). Age-related differences in CYP3A expression and activity in the rat liver, intestine, and kidney. *Journal of Pharmacology and Experimental Therapeutics*.

[6] Yun KU, Oh SJ, Oh JM (2010). Age-related changes in hepatic expression and activity of cytochrome P450 in male rats. *Archives of Toxicology*.

[7] Wauthier V, Verbeeck RK, Calderon PB (2007). The effect of ageing on cytochrome P450 enzymes: consequences for drug biotransformation in the elderly. *Current Medicinal Chemistry*.

[8] Southorn PA, Powis G (1988). Free radicals in medicine. I. Chemical nature and biologic reactions. *Mayo Clinic Proceedings*.

[9] Rizvi SI, Maurya PK (2007). Alterations in antioxidant enzymes during aging in humans. *Molecular Biotechnology*.

[10] Dröge W (2002). Free radicals in the physiological control of cell function. *Physiological Reviews*.

[11] Ceballos-Picot I, Trivier J-M, Nicole A, Sinet P-M, Thevenin M (1992). Age-correlated modifications of copper-zinc superoxide dismutase and glutathione-related enzyme activities in human erythrocytes. *Clinical Chemistry*.

[12] Behne D, Kyriakopoulos A (2001). Mammalian selenium-containing proteins. *Annual Review of Nutrition*.

[13] Inal ME, Kanbak G, Sunal E (2001). Antioxidant enzyme activities and malondialdehyde levels related to aging. *Clinica Chimica Acta*.

[14] Barja G (2002). Rate of generation of oxidative stress-related damage and animal longevity. *Free Radical Biology and Medicine*.

[15] Gil L, Siems W, Mazurek B (2006). Age-associated analysis of oxidative stress parameters in human plasma and erythrocytes. *Free Radical Research*.

[16] Gillette JR, La Du BN, Mandel HG, Way E (1971). Techniques for studying drug metabolism in vitro. *Fundamentals of Drug Metabolism and Drug Disposition*.

[17] Smith PK, Krohn RI, Hermanson GT (1985). Measurement of protein using bicinchoninic acid. *Analytical Biochemistry*.

[18] Weaver RJ, Thompson S, Smith G (1994). A comparative study of constitutive and induced alkoxyresorufin O-dealkylation and individual cytochrome P450 forms in cynomolgus monkey (*Macaca fascicularis*), human, mouse, rat and hamster liver microsomes. *Biochemical Pharmacology*.

[19] Omura T, Sato R (1964). The carbon monoxide-binding pigment of liver microsomes. I. Evidence for its hemoprotein nature. *The Journal of Biological Chemistry*.

[20] Cashman JR, Hanzlik RP (1981). Microsomal oxidation of thiobenzamide. A photometric assay for the flavin-containing monooxygenase. *Biochemical and Biophysical Research Communications*.

[21] Maté L, Virkel G, Lifschitz A, Ballent M, Lanusse C (2008). Hepatic and extra-hepatic metabolic pathways involved in flubendazole biotransformation in sheep. *Biochemical Pharmacology*.

[22] Palackal NT, Lee SH, Harvey RG, Blair IA, Penning TM (2002). Activation of polycyclic aromatic hydrocarbon trans-dihydrodiol proximate carcinogens by human aldo-keto reductase (AKR1C) enzymes and their functional overexpression in human lung carcinoma (A549) cells. *Journal of Biological Chemistry*.

[23] Habig WH, Pabst MJ, Jakoby WB (1974). Glutathione S transferases. The first enzymatic step in mercapturic acid formation. *Journal of Biological Chemistry*.

[24] Mizuma T, Machida M, Hayashi M, Awazu S (1982). Correlation of drug conjugative metabolism rates between in vivo and in vitro: glucuronidation and sulfation of p-nitrophenol as a model compound in rat. *Journal of Pharmacobio-Dynamics*.

[25] Goth L (1991). A simple method for determination of serum catalase activity and revision of reference range. *Clinica Chimica Acta*.

[26] Flohé L, Günzler WA (1984). Assays of glutathione peroxidase. *Methods in Enzymology*.

[27] Handy DE, Lubos E, Yang Y (2009). Glutathione peroxidase-1 regulates mitochondrial function to modulate redox-dependent cellular responses. *Journal of Biological Chemistry*.

[28] Pérez FJ, Villegas D, Mejia N (2002). Ascorbic acid and flavonoid-peroxidase reaction as a detoxifying system of H_2_O_2_ in grapevine leaves. *Phytochemistry*.

[29] Laemmli UK (1970). Cleavage of structural proteins during the assembly of the head of bacteriophage T4. *Nature*.

[30] Towbin H, Staehelin T, Gordon J (1979). Electrophoretic transfer of proteins from polyacrylamide gels to nitrocellulose sheets: procedure and some applications. *Proceedings of the National Academy of Sciences of the United States of America*.

[31] Farahmand SK, Samini F, Samini M, Samarghandian S (2013). Safranal ameliorates antioxidant enzymes and suppresses lipid peroxidation and nitric oxide formation in aged male rat liver. *Biogerontology*.

[32] Parkinson A, Klaassen CD (2001). Biotransformation of Xenobiotics. *Casarett and Doull’s Toxicology: The Basic Science of Poisons*.

[33] Chengelis CP (1988). Age- and sex-related changes in the components of the hepatic microsomal mixed function oxidase system in Sprague-Dawley rats. *Xenobiotica*.

[34] Wauthier V, Verbeeck RK, Calderon PB (2004). Age-related changes in the protein and mRNA levels of CYP2E1 and CYP3A isoforms as well as in their hepatic activities in Wistar rats. What role for oxidative stress?. *Archives of Toxicology*.

[35] Schmucker DL (2001). Liver function and phase I drug metabolism in the elderly: a paradox. *Drugs and Aging*.

[36] Handler JA, Brian WR (1997). Effect of aging on mixed-function oxidation and conjugation by isolated perfused rat livers. *Biochemical Pharmacology*.

[37] Burke MD, Thompson S, Weaver RJ, Wolf CR, Mayer RT (1994). Cytochrome P450 specificities of alkoxyresorufin O-dealkylation in human and rat liver. *Biochemical Pharmacology*.

[38] Kobayashi K, Urashima K, Shimada N, Chiba K (2002). Substrate specificity for rat cytochrome P450 (CYP) isoforms: screening with cDNA-expressed systems of the rat. *Biochemical Pharmacology*.

[39] Hines RN, Cashman JR, Philpot RM, Williams DE, Ziegler DM (1994). The mammalian flavin-containing monooxygenases: molecular characterization and regulation of expression. *Toxicology and Applied Pharmacology*.

[40] Janmohamed A, Hernandez D, Phillips IR, Shephard EA (2004). Cell-, tissue-, sex- and developmental stage-specific expression of mouse flavin-containing monooxygenases (Fmos). *Biochemical Pharmacology*.

[41] Zhang J, Cashman JR (2006). Quantitative analysis of FMO gene mRNA levels in human tissues. *Drug Metabolism and Disposition*.

[42] Okamoto M, Kita T, Okuda H, Tanaka T, Nakashima T (1994). Effects of aging on acute toxicity of nicotine in rats. *Pharmacology and Toxicology*.

[43] Inazu N, Fujii T (1993). Effects of age and calcium ion on testis carbonyl reductase in rats. *Japanese Journal of Pharmacology*.

[44] Tarloff JB, Goldstein RS, Sozio RS, Hook JB (1991). Hepatic and renal conjugation (phase II) enzyme activities in young adult, middle-aged, and senescent male Sprague-Dawley rats. *Proceedings of the Society for Experimental Biology and Medicine*.

[45] Boušová I, Skálová L (2012). Inhibition and induction of glutathione S-transferases by flavonoids: possible pharmacological and toxicological consequences. *Drug Metabolism Reviews*.

[46] Helmy MM (2012). Potential hepato-protective effect of a-tocopherol or simvastatin in aged rats. *Pharmacological Reports*.

[47] Chengelis CP (1988). Age- and sex-related changes in epoxide hydrolase, UDP-glucuronosyl transferase, glutathione S-transferase, and PAPS sulphotransferase in Sprague-Dawley rats. *Xenobiotica*.

[48] Aydin AF, Küçükgergin C, Özdemirler-Erata G, Koçak-Toker N, Uysal M (2010). The effect of carnosine treatment on prooxidant-antioxidant balance in liver, heart and brain tissues of male aged rats. *Biogerontology*.

[49] Carrillo M-C, Nokubo M, Kitani K, Satoh K, Sato K (1991). Age-related alterations of enzyme activities and subunits of hepatic glutathione S-transferases in male and female Fischer-344 rats. *Biochimica et Biophysica Acta*.

[50] Kim H-G, Hong S-M, Kim S-J (2003). Age-related changes in the activity of antioxidant and redox enzymes in rats. *Molecules and Cells*.

[51] Oztürk O, Gümüşlü S (2004). Changes in glucose-6-phosphate dehydrogenase, copper, zinc-superoxide dismutase and catalase activities, glutathione and its metabolizing enzymes, and lipid peroxidation in rat erythrocytes with age. *Experimental Gerontology*.

[52] Maurya PK, Rizvi SI (2010). Age-dependent changes in glutathione-S-transferase: correlation with total plasma antioxidant potential and red cell intracellular glutathione. *Indian Journal of Clinical Biochemistry*.

[53] Kassner N, Huse K, Martin H-J (2008). Carbonyl reductase 1 is a predominant doxorubicin reductase in the human liver. *Drug Metabolism and Disposition*.

[54] Klaunig JE, Kamendulis LM, Hocevar BA (2010). Oxidative stress and oxidative damage in carcinogenesis. *Toxicologic Pathology*.

[55] Ji LL, Dillon D, Wu E (1990). Alteration of antioxidant enzymes with aging in rat skeletal muscle and liver. *The American Journal of Physiology*.

[56] Mármol F, Sánchez J, López D (2010). Role of oxidative stress and adenosine nucleotides in the liver of aging rats. *Physiological Research*.

